# Association between sex hormone binding globulin and metabolic syndrome in US adults: insights from National Health and Nutrition Examination Survey (NHANES) 2013–2016

**DOI:** 10.1186/s13098-024-01398-6

**Published:** 2024-07-18

**Authors:** Yang Yang, Jie Wang, Yi Huang, Yuhang Liu, Shuwan Liu, Huabao Liu, Meiao Tan

**Affiliations:** 1 Department of Liver, Chongqing Traditional Chinese Medicine Hospital, Chongqing, 400000 China; 2 Xiyuan Hospital, China Academy of Chinese Medical Sciences, Beijing, 100000 China

**Keywords:** Sex hormone binding globulin, NHANES, Metabolic syndrome, Cross-sectional study

## Abstract

**Background:**

Metabolic syndrome (MetS) presents a notable public health challenge on a global scale, exerting a considerable impact on individuals’ health and quality of life. There is mounting evidence indicating a robust association between MetS and levels of sex hormones. Therefore, the study aims to explore the relationship between sex hormone binding-globulin (SHBG) and MetS, and to provide evidence that could inform the development of effective prevention strategies for MetS.

**Methods:**

Data for this cross-sectional investigation were collected during the 2013–2016 cycle of the National Health and Nutrition Examination Survey (NHANES), from which 5,499 adults were sampled. The criteria established by the Adult Treatment Program III of the National Cholesterol Education Program were utilized to define MetS. SHBG levels were measured using a standardized technique. Multivariate-adjusted logistic regression, multivariate restricted cubic spline, and threshold effect analyses were utilized to investigate the association between SHBG levels and MetS. Moreover, the stratified analyses and interaction tests of covariables were presented in a forest plot. Finally, sensitivity analysis was utilized to ensure the robustness of the results.

**Results:**

Overall, 1822 participants had MetS. After adjusting for possible confounders, SHBG levels were associated with MetS (Odds ratio [OR], 0.984; 95% confidence interval [CI], 0.981–0.986; *P* < 0.01). The multivariate restricted cubic spline analysis demonstrated a non-linear association between SHBG and MetS (*P* < 0.001). With two piecewise regression models, the adjusted OR of developing MetS was 0.964 (95% CI, 0.959–0.969; *P* < 0.001) among people with SHBG < 76.653 nmol/L, but there was no correlation between SHBG and MetS in participants with SHBG ≥ 76.653 nmol/L. The stability of the association between SHBG levels and MetS was confirmed using subgroup analysis and sensitivity analyses.

**Conclusions:**

Our results suggest that reduced SHBG levels are associated with an increased prevalence of MetS in adults, particularly when SHBG levels are below 76.653 nmol/L. More investigation is required to understand comprehend the mechanisms underlying these results and to delve into their clinical implications.

**Supplementary Information:**

The online version contains supplementary material available at 10.1186/s13098-024-01398-6.

## Introduction

Metabolic syndrome (MetS) involves the coexistence of several risk factors stemming from metabolic and cardiovascular origins, which collectively contribute to its manifestation. These risk factors share common underlying causal mechanisms, resulting in a complex interplay that characterizes MetS [1]. Numerous studies have been conducted to investigate the definition, prevalence, and related features of MetS, as well as to explore its connection with cardiovascular disease (CVD) [2], diabetes mellitus (DM) [3], and dementia [4]. MetS is widely widespread in several nations such as the United States [5], China [6], and is associated with increased all-cause mortality [7]. Despite variations in the definitions of MetS, it is widely acknowledged as a significant health-related condition. Due to the significant health risks associated with MetS, there is considerable scholarly focus on identifying methods for early detection and intervention in related fields.

Obesity and MetS represent significant health challenges in the U.S, and they are associated with disturbances in sex hormone regulation [8]. Human sex hormone-binding globulin (SHBG) is a serum protein with a strong and specific ability to bind to androgens and estrogens [9]. Research has revealed that the prevalence of low SHBG levels in the American population is 3.3% in males and 2.7% in females. Risk factors associated with low SHBG levels include elevated body mass index, diabetes, race (Hispanic, non-Hispanic black, or non-Hispanic white), chronic obstructive pulmonary disease, coronary heart disease, smoking and exposure to phthalates [8, 10]. SHBG levels are independently associated with the risk of diabetes, dementia, non-alcoholic fatty liver disease, hypertension, coronary heart disease, and ischemic stroke [11–14], all of which are associated with a higher risk of MetS.

Furthermore, a study proposed that SHBG might serve as a promising therapeutic option for liver metabolic disorders [15]. Some studies have indicated that SHBG can improve lipid metabolism [16], and enhance insulin sensitivity [17]. While some researches has explored the relationship between MetS and SHBG levels [18–23], the association between SHBG and MetS remains a topic of debate. The consensus on this matter is based on low-quality evidence and lacks definitive data, failing to explore the dose-response relationship between SHBG levels and MetS. Hence, our hypothesis was that sex hormones might underlie the observed alterations in insulin resistance and shed light on the factors influencing MetS. Therefore, the main objective of our study was to explore the association between SHBG and MetS, utilizing data from the National Health and Nutrition Examination Survey (NHANES). Through this extensive cross-sectional survey, our research aimed to offer fresh perspectives on the link between SHBG and MetS among American adults.

## Materials and methods

### Study design and population

Our study relied on data from the National Health and Nutrition Examination Survey (NHANES), conducted by the National Center for Health Statistics (NCHS). The NHANES is an extensive survey aimed at gathering detailed information regarding the health and nutritional status of the non-institutionalized civilian population across the United States. NHANES employs a stratified, multistage probability sampling approach to ensure a varied representation, recruiting participants from various regions throughout the nation. Data collection for the survey involves standardized in-home interviews, physical examinations, and laboratory tests conducted at mobile examination facilities [24]. Since 1999, the survey has been carried out, with updated data sets made accessible every two years at https://www.cdc.gov/nchs/nhanes/index.htm [25]. All survey participants supplied informed written agreement, which was approved ethically by the Ethics Review Board of the NCHS [26].

**Fig. 1:**
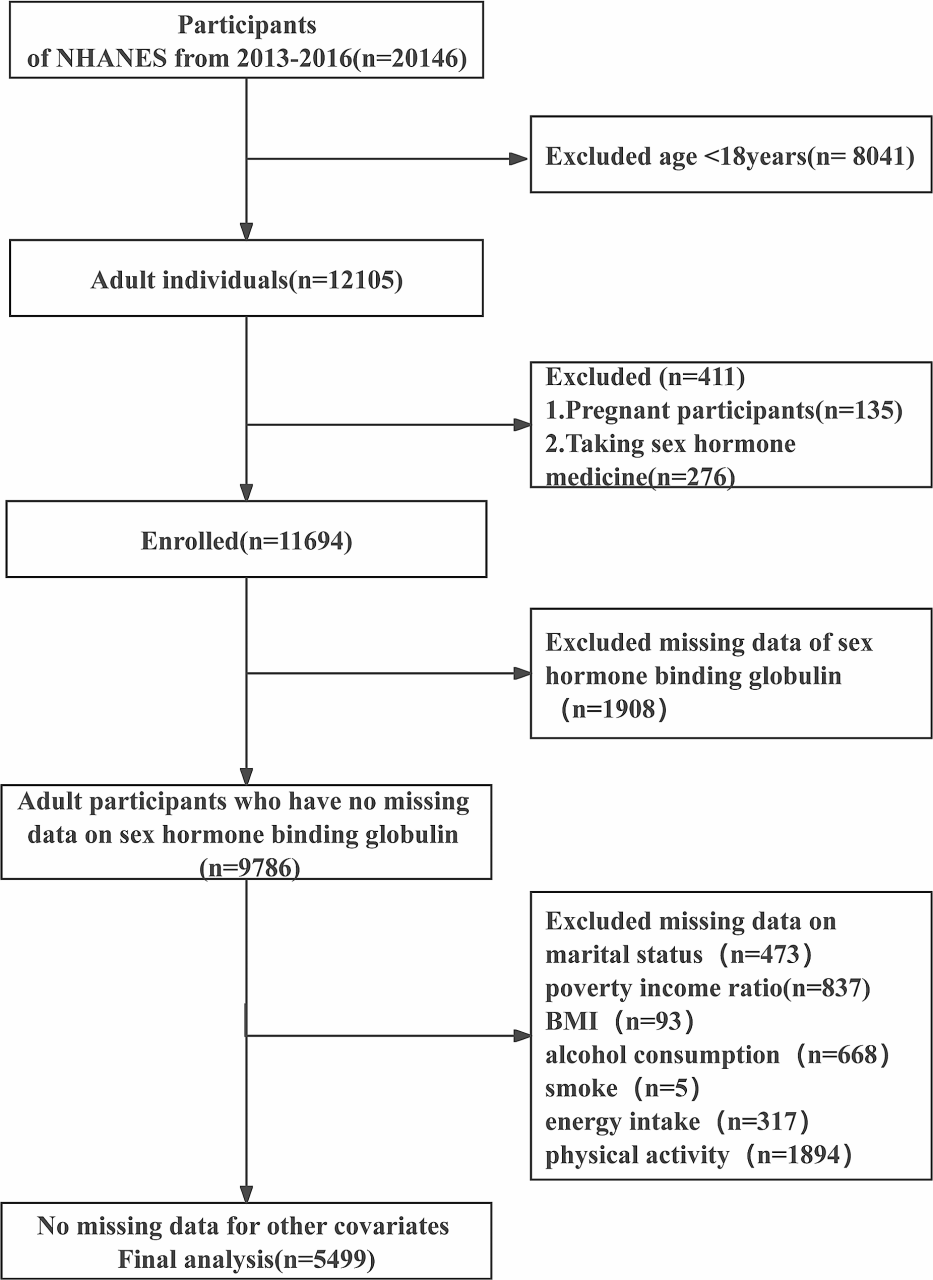
Inclusion and exclusion process for the final analysis was based on the 2013–2016 National Health and Nutrition Examination Survey

In this study, we focused on adults who participated in the 2013–2016 NHANES cycle. The original sample comprised 20,146 participants. We excluded individuals under 18 years of age, pregnant women, those taking sex hormone medication, and individuals with missing data on SHBG and certain covariates (such as marital status, poverty income ratio (PIR), body mass index (BMI), alcohol consumption, smoking, energy intake, and physical activity). Ultimately, the study included 5499 participants. The flowchart depicting the sample selection is shown in Fig. 1.

### Assessment of sex hormone binding globulin

In this study, SHBG, a glycoprotein that binds to testosterone and estradiol, was measured in blood samples using immuno-antibodies and chemiluminescence. The limit of detection for SHBG was determined to be 0.80 nmol/L [27].

### Metabolic syndrome definition

MetS diagnosis follows the criteria specified in the Adult Treatment Program III of the National Cholesterol Education Program. The criteria consist of the following: (1) Triglyceride (TG) levels ≥ 1.69 mmol/L (150 mg/dL); (2) Reduced levels of high-density lipoprotein cholesterol (HDL-C): < 1.03 mmol/L (40 mg/dL) in men and < 1.29 mmol/L (50 mg/dL) in women; (3) Elevated fasting plasma glucose (FPG) levels ≥ 6.1 mmol/L (110 mg/dL); (4) Increased waist circumference (WC): > 102 cm in men and > 88 cm in women; (5) Systolic blood pressure equal to or greater than 140 mmHg and/or diastolic blood pressure equal to or greater than 90 mmHg. Blood samples were obtained in the morning after a 9-hour fast, and blood pressure was measured three times by the physician to establish the average value [28].

### Covariates

Drawing from prior research [28], we incorporated covariates for MetS such as low socioeconomic status, smoking habits, alcohol consumption, physical activity level, energy intake, and family history of diabetes. We accounted for various demographic and lifestyle factors in our analysis, including sex (male or female), age (categorized as 18–39, 40–59, or ≥ 60 years), race/ethnicity (non-Hispanic black, Mexican American, non-Hispanic white, or other races), marital status (married, single, or separated), educational attainment (less than high school, high school, or more than high school), poverty income ratio (categorized as < 1.3 or ≥ 3.5), smoking status (never smoked, ever smoked but quit before the survey, or current smoker), body mass index (BMI) and self-reported family history of diabetes. According to the definition of alcohol consumption in previous literature [29], participants were categorized into four groups: (a) Never drinking: individuals with no history of alcohol consumption or former drinkers, (b) Current heavy drinking (≥ 3 drinks/day for women, ≥ 4 drinks/day for men), (c) Current moderate drinking (≥ 2 drinks/day for women, ≥ 3 drinks/day for men), (d) Current light drinking: individuals who do not meet the criteria for the above categories. Additionally, we incorporated covariates representing factors associated with adverse cardiometabolic health risks [30], such as physical activity, and energy intake. According to the literature [31], physical activity was transformed into metabolic equivalent (MET) minutes of moderate to vigorous physical activity per week. Based on this literature [32], dietary intake was calculated. Due to the fact that SHBG changes over time, we included the time of blood draw as a covariate [27]. A comprehensive categorization of these factors is presented in Table 1.

**Table 1:** Baseline characteristics of participants with and without MetS

Variable	Total (*n* = 5499)	No MetS (*n* = 3677)		MetS (*n* = 1822)	*p*
Age, year	47.8 ± 17.0	45.0 ± 17.1		53.4 ± 15.3	< 0.001
Gender, *n* (%)					0.002
Male	2966 (53.9)	2038 (55.4)		928 (50.9)	
Female	2533 (46.1)	1639 (44.6)		894 (49.1)	
Race, *n* (%)					< 0.001
Non-Hispanic White	2264 (41.2)	1508 (41.0)	756 (41.5)		
Non-Hispanic Black	1072 (19.5)	742 (20.2)	330 (18.1)		
Mexican American	812 (14.8)	491 (13.4)	321 (17.6)		
Others	1351 (24.6)	936 (25.5)	415 (22.8)		
Marital status, *n* (%)					0.002
Married	3340 (60.7)	2181 (59.3)	1159 (63.6)		
Single or separated	2159 (39.3)	1496 (40.7)	663 (36.4)		
PIR, (%)					< 0.001
< 1.3	1680 (30.6)	1078 (29.3)	602 (33.0)		
1.3–3.5	2053 (37.3)	1356 (36.9)	697 (38.3)		
>= 3.5	1766 (32.1)	1243 (33.8)	523 (28.7)		
Educational level, *n* (%)					< 0.001
Less than high school	971 (17.7)	596 (16.2)	375 (20.6)		
High school diploma	1244 (22.6)	797 (21.7)	447 (24.5)		
More than high school	3284 (59.7)	2284 (62.1)	1000 (54.9)		
Smoking, *n* (%)					< 0.001
Never	3083 (56.1)	2107 (57.3)	976 (53.6)		
Former	1099 (20.0)	752 (20.5)	347 (19.0)		
Now	1317 (23.9)	818 (22.2)	499 (27.4)		
Alcohol consumption, *n* (%)					< 0.001
Former	814 (14.8)	432 (11.7)	382 (21.0)		
Heavy	1130 (20.5)	807 (21.9)	323 (17.7)		
Mild	1917 (34.9)	1315 (35.8)	602 (33.0)		
Moderate	898 (16.3)	653 (17.8)	245 (13.4)		
Never	740 (13.5)	470 (12.8)	270 (14.8)		
BMI, (kg/m^2^)	29.2 ± 6.9	27.3 ± 6.2	33.1 ± 6.7		< 0.001
Total Energy intake (kcal)	2018.0 (1492.5, 2665.0)	2059.0 (1512.0, 2698.0)	1963.0 (1461.0, 2587.5)		< 0.001
Total PA MET (minutes/week)	2160.0 (840.0, 6000.0)	2400.0 (960.0, 6720.0)	1680.0 (610.0, 4800.0)		< 0.001
SHBG, (nmol/L)	46.3 (31.4, 68.9)	50.2 (34.0, 75.5)	39.2 (27.5, 57.0)		< 0.001
Time of blood draw, *n* (%)					0.133
Morning	2618 (47.6)	1718 (46.7)	900 (49.4)		
Afternoon	886 (16.1)	611 (16.6)	275 (15.1)		
Evening	1995 (36.3)	1348 (36.7)	647 (35.5)		
Family history of diabetes, *n*(%)					0.070
No	4360 (79.3)	2941 (80.0)	1419 (77.9)		
Yes	1139 (20.7)	736 (20.0)	403 (22.1)		

Data are presented as mean (SD) or n (%). MetS: Metabolic Syndrome; SHBG: sex hormone binding globulin; PIR: poverty income ratio; BMI: body mass index; PA: physical activity

### Statistical analysis

All participants were categorized into two groups based on the presence or absence of MetS. Continuous variables were reported as either mean ± standard deviation for normally distributed data, or as medians and interquartile ranges (IQR, 25–75%) for abnormally distributed data, while categorical variables were presented as frequencies (percentages).

To assess the linear correlation between SHBG levels and MetS, we utilized restricted cubic spline (RCS) analysis. Additionally, we conducted a multivariate Logistic regression analysis using different models to explore the relationship between SHBG levels and the risk of developing MetS. In Model 1, we adjusted for age and sex. In Model 2, we extended our adjustments to incorporate additional variables including educational level, PIR, and marital status. In Model 3, we included additional variables beyond those in Model 2 to enhance the scope of our analysis, including BMI, alcohol consumption, smoking, physical activity, family history of diabetes and energy intake.

Additionally, subgroup analyses were conducted based on age (< 40 years, ≥ 60 years), sex (male, female), PIR (< 1.3, > 3.5), BMI (< 25, ≥ 30), smoking status (never, former, current), and alcohol consumption (former, current, mild, moderate, heavy), as well as family history of diabetes to explore the association between SHBG levels and the risk of MetS.

Multivariate Logistic regression was employed to evaluate diversity within subgroups, with interactions between subgroups scrutinized via likelihood ratio testing. To fortify the reliability of our results, we conducted sensitivity analyses by employing multiple imputation to address missing values in covariates. Furthermore, we analyzed the association between SHBG levels and MetS according to the 2009 criteria outlined by the International Diabetes Federation for defining MetS [33].

Since the sample size was determined solely based on the available data, no preliminary statistical power estimates were carried out. All analyses were performed using the statistical software packages R 4.2.2 (http://www.R-project.org, The R Foundation) and Free Statistics analysis platform (Version 1.9, Beijing, China, http://www.clinicalscientists.cn/freestatistics) [34]. A study was done to describe all participants, and it was found that the p-value was < 0.05, indicating significance in a two-tailed test.

## Result

### Baseline characteristics of participants

Following a thorough screening procedure that adhered to predefined inclusion and exclusion criteria, the study enrolled a total of 5499 patients. Among these subjects, 1822 had a documented history of MetS. The baseline characteristics of the patients were categorized according to the presence or absence of MetS, can be found in Table 1. Participants with reduced levels of SHBG were diagnosed with MetS (*P* < 0.001). Patients with MetS exhibited distinctive features compared to those without MetS. Specifically, the MetS group had a higher proportion of male participants, smokers, and alcohol consumers (*P* < 0.001), as well as lower educational attainment levels and PIR (*P* < 0.001), compared to the non-MetS group. However, no statistically significant differences were detected among the groups concerning to a family history of diabetes (*P* > 0.05).

### Association between sex hormone binding globulin and metabolic syndrome

In the multivariate logistic regression analyses, there was an inverse relationship between SHBG, analyzed as a continuous variable, and the probability of MetS (OR = 0.988, 95% CI = 0.986–0.990, *P* < 0.001). This association remained statistically significant even after adjusting for age, sex, race, educational level, PIR, marital status, BMI, alcohol consumption, smoke, physical activity, energy intake, family history of diabetes (OR = 0.984, 95% CI = 0.981–0.986, *P* < 0.001, Table 2).

**Table 2:** Association between SHBG and MetS among adult participants in the NHANES 2013-2016 cycles

	Crude model		Model 1		Model 2		Model 3	
Variable	OR (95%CI)	*P*	OR (95%CI)	*P*	OR (95%CI)	*P*	OR (95%CI)	*P*
SHBG (nmol/l)	0.988 (0.986 ∼ 0.990)	< 0.001	0.976 (0.973 ∼ 0.979)	< 0.001	0.980 (0.970 ∼ 0.980)	< 0.001	0.984 (0.981 ∼ 0.986)	< 0.001

Crude Model: No adjustment;

Model 1: Age, sex were adjusted;

Model 2: Age, sex, race, educational level, poverty income ratio, marital status were adjusted;

Model 3: Age, sex, race, educational level, poverty income ratio, marital status, BMI, alcohol consumption, smoke, physical activity, energy intake, family history of diabetes;

SHBG: Sex Hormone-Binding Globulin; OR: Odds ratio.

### Analysis of restricted cubic spline regression

**Fig. 2:**
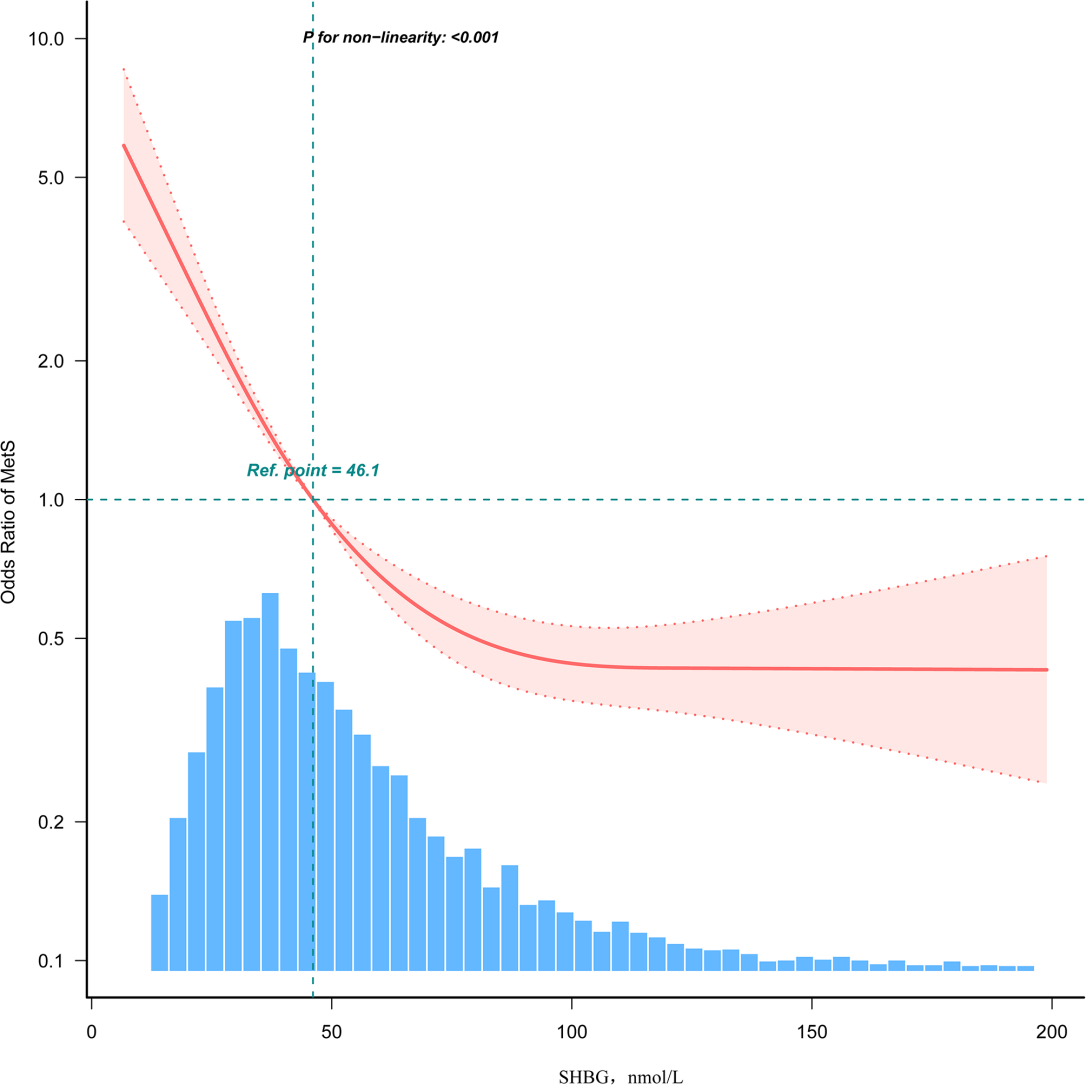
Association between SHBG and MetS. Solid and dashed lines indicate the predicted value and 95% CI. The restricted cubic spline model was adjusted for age, sex, race, educational level, poverty income ratio, marital status, BMI, alcohol consumption, smoking, physical activity, energy intake, family history of diabetes. SHBG, sex hormone binding globulin. BMI, Body mass index

After accounting for several covariates, we identified a significant non-linear relationship between SHBG levels and MetS in the RCS regression analysis (*P* < 0.001; Fig. 2), characterized by an L-shaped dose-response curve. As depicted in Table 3, when SHBG levels were below 76.653 nmol/L, the risk of MetS decreased as SHBG increased (OR = 0.964, 95% CI = 0.959–0.969, *P* < 0.001). Conversely, beyond the turning point of 76.653 nmol/L, the estimated dose-response curve indicated a flat line, suggesting a non-significant association between SHBG levels and the risk of MetS. (Table 2).

**Table 3:** Association between SHBG and MetS using two-piecewise regression models

SHBG	Adjusted Model*	
	OR (95%CI)	*P*
< 76.653nmol/L	0.964 (0.959 ∼ 0.969)	< 0.001
≥ 76.653 nmol/L	1.001 (0.996 ∼ 1.006)	0.763
Likelihood Ratio test		< 0.001

Adjusted for age, sex, race, educational level, poverty income ratio, marital status, BMI, alcohol consumption, smoking, physical activity, energy intake, family history of diabetes

### Stratified analyses based on additional variables

**Fig. 3:**
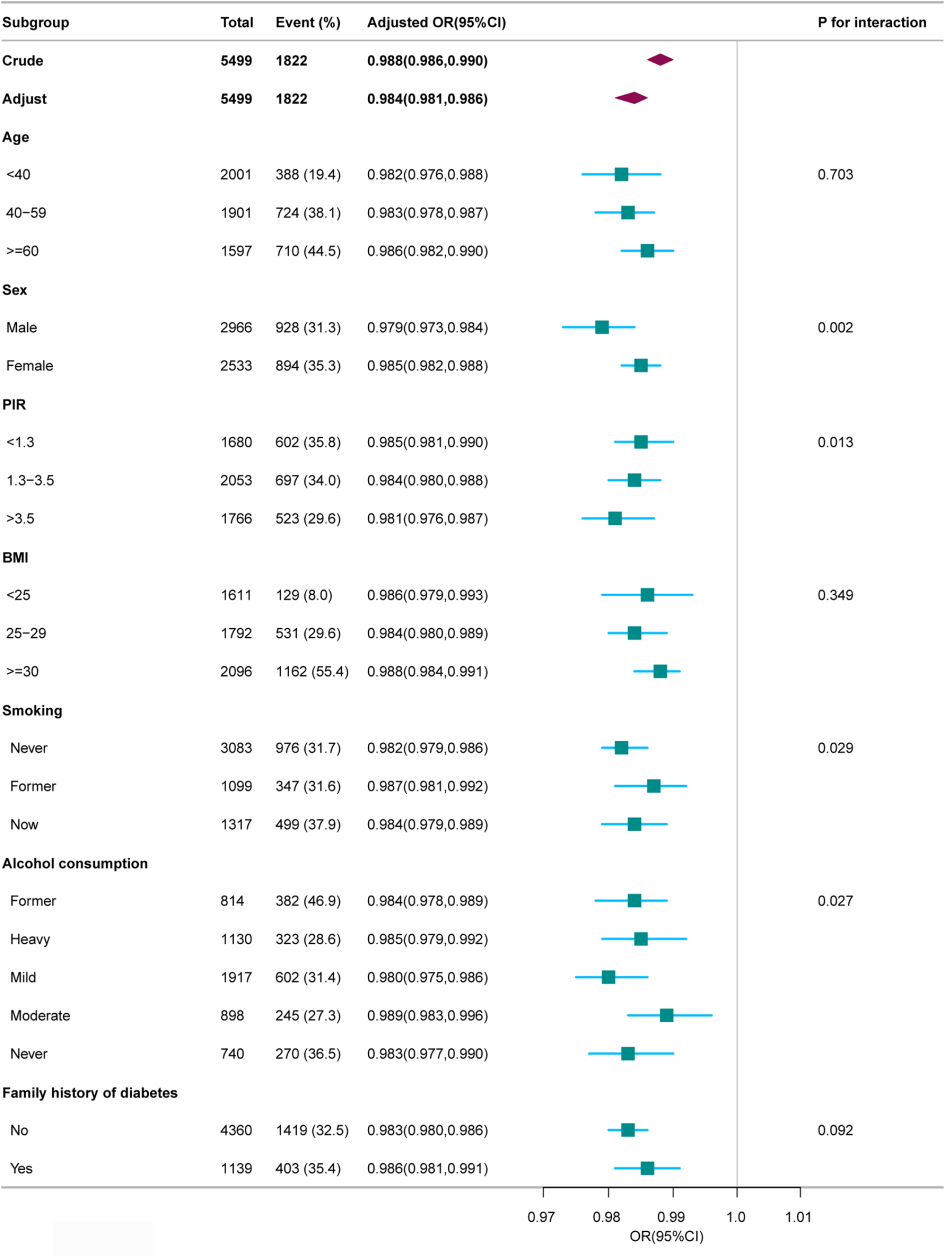
Association between SHBG and MetS Except for the stratification factor itself, the stratifications were adjusted for all variables (age, sex, race, educational level, poverty income ratio, marital status, BMI, alcohol consumption, smoking, physical activity, energy intake, family history of diabetes). BMI, body mass index

We conducted a stratified analysis across several subgroups to explore potential variations in the relationship between SHBG levels and MetS (Fig. 3). Following stratification by age, BMI, and family history of diabetes, no significant interactions were observed in any subgroups. Taking multiple testing into consideration, it’s important to recognize that a *p*-value below 0.05 for interactions involving sex, PIR, smoking, and alcohol consumption may not reach statistical significance.

### Sensitivity analysis

In the sensitivity analysis, SHBG levels were converted from a continuous variable to a categorical variables, and grouped into quartiles (Q1-Q4). The results of the sensitivity analysis suggested that individuals in the Q2, Q3, and Q4 groups had a lower risk of developing MetS compared to those in the Q1 group (Additional file 1: Table S1). Furthermore, we reanalyzed the link between SHBG levels and MetS using the MetS criteria defined by the 2009 International Diabetes Federation, and the results confirmed a stable association between SHBG levels and MetS. (Additional file 1: Table S2). Because of a significant amount of missing data in covariates, we conducted multiple imputation on the covariates and performed multiple model logistic regression, curve fitting, and inflection point analysis on a dataset (Additional file 1: Table S3, Table S4, Fig. S1). The results revealed no significant changes.

## Discussion

Our research utilized data from the NHANES in the United States to assess the correlation between SHBG levels and the prevalence of MetS among adults. Even after accounting for potential confounding factors, high levels of SHBG showed a linked reduction in the risk of developing MetS. Notably, our findings revealed a non-linear relationship in the form of an “L” shaped curve (P for non-linearity < 0.001), indicating that the relationship between SHBG levels and MetS was notably accentuated at certain thresholds. Specifically, the relationship appeared to plateau at SHBG levels of 76.653 nmol/L. Furthermore, through exploratory subgroup analyses and sensitivity analysis, we observed that the association between SHBG levels and MetS remained consistent in adults.

Numerous studies have indicated an association between SHBG levels and MetS [18, 35–37]. However, the association between SHBG levles and MetS remains unclear. The previous studies have presented conflicting findings, highlighting the need for further investigation in this area. In the context of male, a study revealed a statistically significant link between decreased SHBG levels and a higher incidence rate of MetS [18]. Meanwhile, in studies involving women, independent inverse correlations between SHBG and MetS were identified [38]. Nevertheless, research indicated that the association between SHBG levels and MetS is not statistically significant among postmenopausal women [20]. Similarly, another study revealed that SHBG levels is not associated with MetS [21]. Notably, a meta-analysis showed a negative association between SHBG levels and MetS, with no gender difference [22]. In conclusion, total SHBG levels has been found to be inversely associated with incident MetS in men. However, none of the studies indicated the critical value of the correlation between SHBG levels and MetS, whereas our study used statistical methods such as restricted cubic splines and logistic regression inflection point analysis to observe that there was no linear association between SHBG levels and the occurrence of MetS.

SHBG is secreted by liver cells and can bind to steroid hormones such as testosterone and estradiol, regulating their bioavailability and delivery to target organs and tissues [15].The Free active testosterone concentrations in plasma are substantially impacted by SHBG concentrations, as 65% of free active testosterone is bound to SHBG. Consequently, adults with low SHBG can have elevated bioavailable and free testosterone levels [39]. Elevated testosterone levels may reduce the incidence rate of MetS [40, 41]. A recent study found a positive association between the free estradiol index and the incidence of MetS in males of all ages, as well as in older females [23]. Furthermore, lower SHBG levels were associated with higher daily alcohol intake levels, higher BMI, and a higher risk of diabetes, CHD, and non-alcoholic fatty liver disease [37, 42–47], - all of which contributed to elevated risk of MetS.

The investigation carried out by our team has numerous notable strengths. Firstly, we utilized a large representative sample from the NHANES database, which enhances the generalizability and applicability of our findings to non-institutionalized civilian populations. Moreover, we established stringent participant selection criteria, that specifically excluded pregnant individuals, those undergoing sex hormone therapy, and those with missing information on SHBG levels and MetS, thereby bolstering the study’s reliability. Furthermore, the extensive sample size enabled us to perform subgroup analyses, thereby allowing us to evaluate the potential impact of additional variables on the association between SHBG levels and MetS.

Nevertheless, this study had several limitations. Primarily, the cross-sectional design hindered the ability to establish causality. Additionally, the assessment of SHBG may have been affected by multiple factors, such as laboratory protocols. Furthermore, although we controlled for numerous potential confounding variables, we were unable to fully mitigate the influence of unmeasured confounders. As a result, it is important to be cautious in drawing conclusions, and additional research in various disease groups is necessary to bolster our findings. For future studies, it is advisable to utilize longitudinal study designs to investigate the possible causal connection between SHBG levels and the development of MetS. Finally, it is important to note that the NHANES database only provides data on the United States population. Therefore, further studies are needed to corroborate the linkage between SHBG and MetS in various national populations. Moreover, delving into the genetic correlations between SHBG levels and the occurrence of MetS would further advance our comprehension of this relationship.

## Conclusions

This study aimed to assess the association between SHBG levels and MetS. Higher levels of SHBG were inversely related to MetS in adults, even after adjusting for other potential confounding factors. There was a non-linear L-shaped association between SHBG levels and MetS, with a threshold value of 76.653 nmol/L. These results are noteworthy and can have implications for healthcare providers treating MetS. However, due to the potential for confounding factors, further research is necessary to confirm the findings.

### Electronic supplementary material

Below is the link to the electronic supplementary material.

Supplementary Material 1

Supplementary Material 2

Supplementary Material 3

Supplementary Material 4

## Data Availability

No datasets were generated or analysed during the current study.

## Abbreviations

BMI: Body mass index
BP: Blood pressure
FPG: Fasting plasma glucose
MetS: Metabolic syndrome
NCHS: National Center for Health Statistics
NHANES: National Health and Nutrition Examination Survey
OR: Odds ratio
PIR: Poverty income ratio
SHBG: Sex hormone-binding globulin

## References

[1] Silveira Rossi JL, Barbalho SM, Reverete de Araujo R, Bechara MD, Sloan KP, Sloan LA (2022). Metabolic syndrome and cardiovascular diseases: going beyond traditional risk factors. Diabetes Metab Res Rev.

[2] Mottillo S, Filion KB, Genest J, Joseph L, Pilote L, Poirier P (2010). The metabolic syndrome and cardiovascular risk a systematic review and meta-analysis. J Am Coll Cardiol.

[3] Li W, Wang D, Wang X, Gong Y, Cao S, Yin X (2019). The association of metabolic syndrome components and diabetes mellitus: evidence from China national stroke screening and prevention project. BMC Public Health.

[4] Qureshi D, Collister J, Allen NE, Kuźma E, Littlejohns T (2024). Association between metabolic syndrome and risk of incident dementia in UK Biobank. Alzheimer’s Dement: J Alzheimer’s Assoc.

[5] Wastyk HC, Perelman D, Topf M, Fragiadakis GK, Robinson JL, Sonnenburg JL (2023). Randomized controlled trial demonstrates response to a probiotic intervention for metabolic syndrome that may correspond to diet. Gut Microbes.

[6] Gu D, Reynolds K, Wu X, Chen J, Duan X, Reynolds RF (2005). Prevalence of the metabolic syndrome and overweight among adults in China. Lancet (Lond Engl).

[7] Li W, Chen D, Peng Y, Lu Z, Kwan M-P, Tse LA (2023). Association between metabolic syndrome and mortality: prospective cohort study. JMIR Public Health Surveillance.

[8] Zhang J, Gu W, Zhai S, Liu Y, Yang C, Xiao L (2024). Phthalate metabolites and sex steroid hormones in relation to obesity in US adults: NHANES 2013–2016. Front Endocrinol.

[9] Thaler MA, Seifert-Klauss V, Luppa PB (2015). The biomarker sex hormone-binding globulin - from established applications to emerging trends in clinical medicine. Best Pract Res Clin Endocrinol Metab.

[10] Wang Y (2021). Definition, prevalence, and risk factors of low sex hormone-binding globulin in US adults. J Clin Endocrinol Metab.

[11] Li N, Huang C, Lan B, Lin D, Wang C, You L (2021). Association of gonadal hormones and sex hormone binding globulin with risk of diabetes: a cohort study in middle-aged and elderly Chinese males. Int J Clin Pract.

[12] Huang J, Xu B, Chen X, Yang L, Liu D, Lin J (2024). Sex hormone-binding globulin and risk of incident dementia in middle-aged to older women: results from the UK biobank cohort study. Neuroendocrinology.

[13] Luo J, Chen Q, Shen T, Wang X, Fang W, Wu X (2018). Association of sex hormone-binding globulin with nonalcoholic fatty liver disease in Chinese adults. Nutr Metab.

[14] Yang Q, Li Z, Li W, Lu L, Wu H, Zhuang Y (2019). Association of total testosterone, free testosterone, bioavailable testosterone, sex hormone-binding globulin, and hypertension. Medicine.

[15] Bourebaba N, Ngo T, Śmieszek A, Bourebaba L, Marycz K (2022). Sex hormone binding globulin as a potential drug candidate for liver-related metabolic disorders treatment. Biomed Pharmacother.

[16] Bourebaba L, Kępska M, Qasem B, Zyzak M, Łyczko J, Klemens M (2023). Sex hormone-binding globulin improves lipid metabolism and reduces inflammation in subcutaneous adipose tissue of metabolic syndrome-affected horses. Front Mol Biosci.

[17] Bourebaba N, Sikora M, Qasem B, Bourebaba L, Marycz K (2023). Sex hormone-binding globulin (SHBG) mitigates ER stress and improves viability and insulin sensitivity in adipose-derived mesenchymal stem cells (ASC) of equine metabolic syndrome (EMS)-affected horses. Cell Commun Signal: CCS.

[18] Li C, Ford ES, Li B, Giles WH, Liu S (2010). Association of testosterone and sex hormone-binding globulin with metabolic syndrome and insulin resistance in men. Diabetes Care.

[19] Fenske B, Kische H, Gross S, Wallaschofski H, Völzke H, Dörr M (2015). Endogenous androgens and sex hormone-binding globulin in women and risk of metabolic syndrome and type 2 diabetes. J Clin Endocrinol Metab.

[20] Hajamor S, Després J-P, Couillard C, Lemieux S, Tremblay A, Prud’homme D (2003). Relationship between sex hormone-binding globulin levels and features of the metabolic syndrome. Metabolism.

[21] Alinezhad A, Jafari F (2019). The relationship between components of metabolic syndrome and plasma level of sex hormone-binding globulin. Eur j Transl Myol.

[22] Brand JS, van der Tweel I, Grobbee DE, Emmelot-Vonk MH, van der Schouw YT (2011). Testosterone, sex hormone-binding globulin and the metabolic syndrome: a systematic review and meta-analysis of observational studies. Int J Epidemiol.

[23] Dubey P, Singh V, Venishetty N, Trivedi M, Reddy SY, Lakshmanaswamy R (2024). Associations of sex hormone ratios with metabolic syndrome and inflammation in US adult men and women. Front Endocrinol.

[24] Liang J, Peng Q, Yang X, Yang C (2021). The association between serum testosterone levels and metabolic syndrome among women. Diabetol Metab Syndr.

[25] Xiong L, Yang G, Guo T, Zeng Z, Liao T, Li Y (2023). 17-year follow-up of association between telomere length and all-cause mortality, cardiovascular mortality in individuals with metabolic syndrome: results from the NHANES database prospective cohort study. Diabetol Metab Syndr.

[26] Fan H, Wang Y, Ren Z, Liu X, Zhao J, Yuan Y (2023). Mediterranean diet lowers all-cause and cardiovascular mortality for patients with metabolic syndrome. Diabetol Metab Syndr.

[27] Guo M, Zhu C (2023). Associations between exposure to a mixture of phenols and sex steroid hormones among pre- and postmenopausal women: evidence from NHANES 2015–2016. Environ Sci Pollut Res Int.

[28] Zhao Y, Shao W, Zhu Q, Zhang R, Sun T, Wang B (2023). Association between systemic immune-inflammation index and metabolic syndrome and its components: results from the national health and nutrition examination survey 2011–2016. J Transl Med.

[29] Li Y, Xiong B, Zhu M, Ren Y, Lan Y, Hu T (2023). Associations of starchy and non-starchy vegetables with risk of metabolic syndrome: evidence from the NHANES 1999–2018. Nutr Metab.

[30] Shakya S, Shrestha V, Neupane D (2023). Social determinants of health and cardiometabolic risk factors in Nepal: a scoping review. Nutr Metab Cardiovasc Dis: NMCD.

[31] Liang J, Huang S, Jiang N, Kakaer A, Chen Y, Liu M (2023). Association between joint physical activity and dietary quality and lower risk of depression symptoms in US adults: cross-sectional NHANES study. JMIR Public Health Surveillance.

[32] Xiang L, Wu M, Wang Y, Liu S, Lin Q, Luo G (2023). Inverse J-shaped relationship of dietary carbohydrate intake with serum klotho in NHANES 2007–2016. Nutrients.

[33] Alberti KGMM, Eckel RH, Grundy SM, Zimmet PZ, Cleeman JI, Donato KA (2009). Harmonizing the metabolic syndrome: a joint interim statement of the international diabetes federation task force on epidemiology and prevention; national heart, lung, and blood institute; American heart association; world heart federation; international atherosclerosis society; and international association for the study of obesity. Circulation.

[34] Liu H, Wang L, Chen C, Dong Z, Yu S (2022). Association between dietary niacin intake and migraine among American adults: national health and nutrition examination survey. Nutrients.

[35] Yeap BB, Marriott RJ, Antonio L, Raj S, Dwivedi G, Reid CM (2022). Associations of serum testosterone and sex hormone-binding globulin with incident cardiovascular events in middle-aged to older men. Ann Intern Med.

[36] Zhao D, Guallar E, Ouyang P, Subramanya V, Vaidya D, Ndumele CE (2018). Endogenous sex hormones and incident cardiovascular disease in post-menopausal women. J Am Coll Cardiol.

[37] Li J, Zheng L, Chan KHK, Zou X, Zhang J, Liu J (2023). Sex hormone-binding globulin and risk of coronary heart disease in men and women. Clin Chem.

[38] Kim C, Halter JB (2014). Endogenous sex hormones, metabolic syndrome, and diabetes in men and women. Curr Cardiol Rep.

[39] Qu X, Donnelly R (2020). Sex hormone-binding globulin (SHBG) as an early biomarker and therapeutic target in polycystic ovary syndrome. Int J Mol Sci.

[40] Ahmad IH, Mohamed Mostafa ER, Mohammed SA, Shipl W, Soliman AA, Said M (2023). Correlations between serum testosterone and irisin levels in a sample of Egyptian men with metabolic syndrome; (case-control study). Arch Physiol Biochem.

[41] Hedderson MM, Capra A, Lee C, Habel LA, Lee J, Gold EB, et al. Longitudinal changes in sex hormone binding globulin (SHBG) and risk of incident diabetes: the study of women’s health across the nation (SWAN). Diabetes Care. 2024;dc231630. 10.2337/dc23-1630. 10.2337/dc23-1630 PMC10973900 38320264

[42] Tin Tin S, Key TJ, Reeves GK (2021). Alcohol intake and endogenous hormones in pre- and postmenopausal women: findings from the UK biobank. Cancer Epidemiol Biomark Prev: Publ Am Assoc Cancer Res Cosponsored Am Soc Prev Oncol.

[43] Marriott RJ, Murray K, Adams RJ, Antonio L, Ballantyne CM, Bauer DC (2023). Factors associated with circulating sex hormones in men: individual participant data meta-analyses. Ann Intern Med.

[44] Kim D, Manikat R, Cholankeril G, Ahmed A (2024). Endogenous sex hormones and nonalcoholic fatty liver disease in US adults. Liver Int: off J Int Assoc Study Liver.

[45] Zeng Y, Cao S, Yang H (2023). Circulating sex hormone-binding globulin levels and ischemic stroke risk: a mendelian randomization study. Postgrad Med J.

[46] Pan D, Guo J, Su Z, Wang J, Wu S, Guo J (2023). Association of the controlling nutritional status score with all-cause mortality and cancer mortality risk in patients with type 2 diabetes: NHANES 1999–2018. Diabetol Metab Syndr.

[47] Liu Z, Wang Q, Huang H, Wang X, Xu C (2023). Association between serum uric acid levels and long-term mortality of metabolic dysfunction-associated fatty liver disease: a nationwide cohort study. Diabetol Metab Syndr.

