# Supplementary figures and images for: Association between sex hormone binding globulin and metabolic syndrome in US adults: insights from National Health and Nutrition Examination Survey (NHANES) 2013–2016
Source: Diabetol Metab Syndr. 2024 Jul 18;16:170. doi: 10.1186/s13098-024-01398-6 (PMC11256583; doi:10.1186/s13098-024-01398-6)

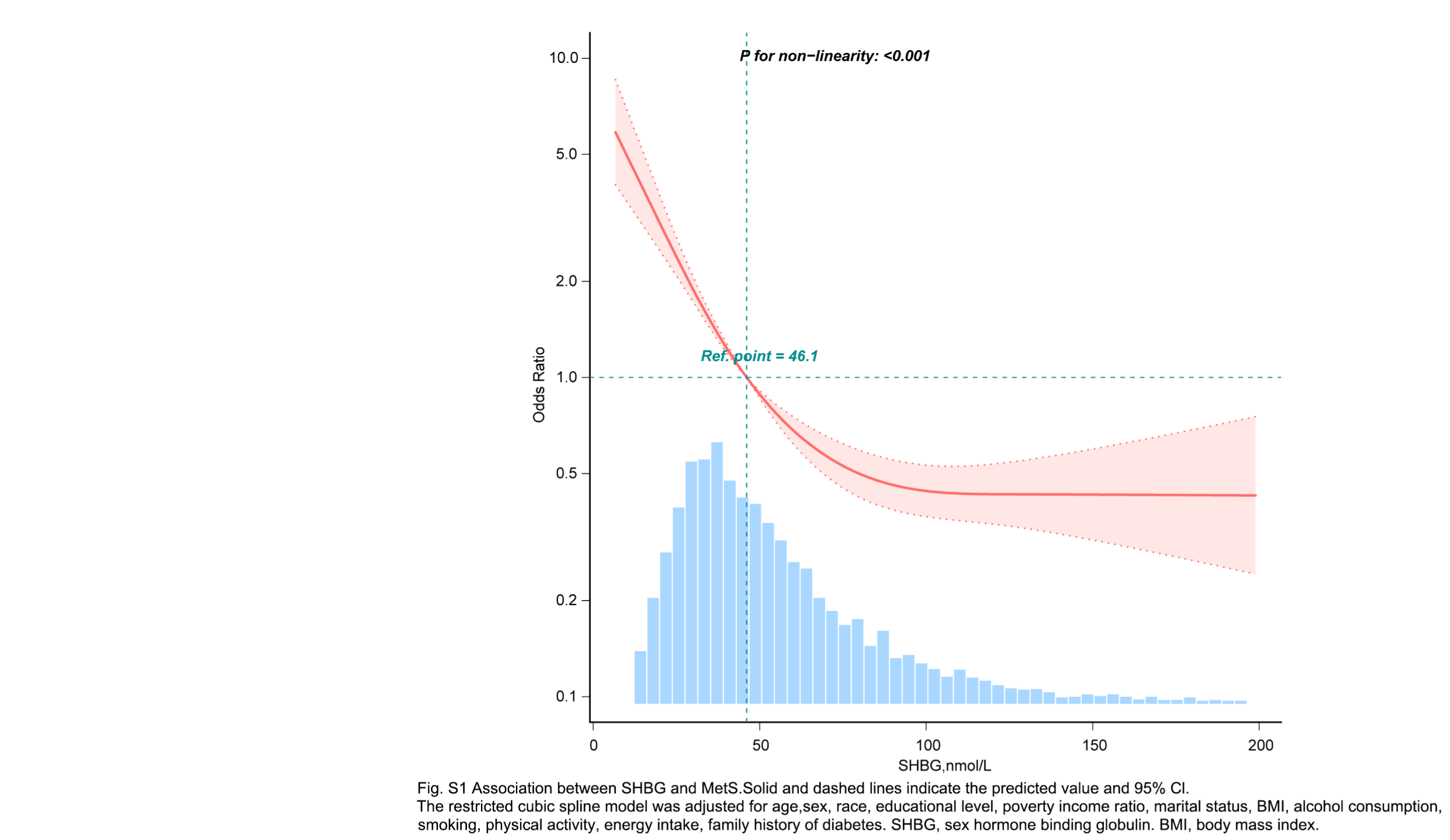

Supplement: Supplementary file 4 — Supplementary Material 4 [file 13098_2024_1398_MOESM4_ESM.tif]
